# An Address on the Improvement of the Medical Profession; Delivered before the Medical Society of Tennessee, at Its Twelfth Annual Meeting, in Nashville, in May, 1841

**Published:** 1841-08

**Authors:** Lunsford P. Yandell

**Affiliations:** Prof. of Chemistry and Pharmacy in the Louisville Medical Institute


### WESTERN JOURNAL

OF

### MEDICINE AND SURGERY.

AUGUST, 1841.

Art. I.—*An Address on the Improvement of the Medical Profession;* delivered before the Medical Society of Tennessee, at its twelfth annual meeting, in Nashville, in May, 1841. By Lunsford P. Yandell, M. D., Prof. of Chemistry and Pharmacy in the Louisville Medical Institute.

It is now ten years since I took leave of this Society as a regular member, and, in resigning the office of Corresponding Secretary, which it had been my honor to hold in it, attempted a sketch of some of the leading objects which such associations are expected to promote. After this lapse of time, it might not be unprofitable, on the present occasion, to enquire how far these objects have been advanced by the labor of the Society. As one of its original members, entertaining a warm personal regard for the individuals who have given it the benefit of their talents, learning and time, and strongly attached to it, also, by feelings of State pride, I might not prove a very impartial witness in the case; but I think even those the least apt to be satisfied, and those the readiest to find fault must admit, that the Medical Society of Tennessee has not existed in vain. I believe the most skeptical and the most censorious will agree, that it has added something to the standing of the medical profession of the State. Instituted for the purpose of rendering the healing art more useful to society and more honorable in itself, one of the objects contemplated by those who framed its constitution, and by those who first met here to organize the Society, was the *suppression of quackery;* and I know that not a few of those who assembled on that occasion have long since withdrawn from the Society, in despair of its ever being able, with its present powers, to accomplish this great work. Failing in what they deemed the chief concern, they have been disposed to look with indifference upon all the other ends for which the Society was created. Disappointed of the treasure for which, with sanguine tempers and stout hearts, they began to dig in this field, they have appeared to contemn the harvest of fame which the Society is gathering for itself and the medical profession in Tennessee. No one would rejoice more heartily than I should at seeing the deep stain of quackery wiped from our profession. It is impossible to look over the world and see how this "foul anomaly" cleaves to medicine in all lands and amongst all people, and not feel pained and humbled on account of the weakness of human nature. But it has long been the conviction of many of the members of this Society, that quackery is not to be suppressed by the strong arm of the law, and least of all in our free Republic. This is not the nation, and this is not the age for monopolies. The spirit of the people and of the times is averse to prescriptive privileges. Laws to restrain empiricism, could they be enacted, would be enforced only when they were in accordance with public sentiment. Opposed to it, or in advance of it, they would be a dead letter. Men will employ those whom they fancy possessed of the skill to relieve their sufferings and prolong their lives, and while constituted as they are, they seem fated to be the dupes of the boastful pretender. Laws do not avail in a case like this. There are rights which men cannot be made to surrender, and although civil law might deprive the empiric of many of the privileges of the educated physician, the sympathies of the people, awakened for one who seemed to be downtrodden by the privileged, would amply compensate him for all that he had been obliged to surrender to the claims of the civil magistrate. The remedy, therefore, would seem to lie, not with the framers of our laws, although a wise legislation might do much for Medical science, but with the profession itself, who must rid society of empiricism by improving the healing art—by making all the professors of skill in it truly skilful—by wiping away all *opprobria medicorum*—by rendering the cure of disease safe, certain, and agreeable.

If it be objected that in this a task is proposed which is above all human power—that there must remain, to the end of time, imperfections in the healing art—diseases which no remedies can reach—diseases growing out of that imperfect organization which bad indulgences have entailed upon the race, my reply is, that so long as our systems of cure remain discordant and our remedies uncertain, so long as there continue to be maladies that defy the resources of enlightened art, we must make up our mind to see empiricism flourish; for so long will there be found men loud and impudent in professions of catholicons and panaceas. Hope springs eternal in the human breast, and the victim of phthisis, just dropping into the grave, will eagerly catch at every "pulmonary balsam" that promises to restore his wasted energies and turn back the ebbing current.

It has, therefore, always appeared to me a great mistake in those members who have abandoned the Society because its charter gives it no power to regulate the practice of physic, and to compel students of medicine, like students of law, to pass a satisfactory examination before assuming its high responsibilities. Most devoutly must every philanthropist wish that such a regulation could be enforced, and that those only were allowed to become the guardians of the public health, who, by a diligent course of study and the use of all the lights afforded to the medical student, have prepared themselves thoroughly for so sacred an office. But no such requisition is made by the statutes of Tennessee; in very few of the States does any such regulation obtain, and where it has an existence under forms of government more energetic than ours, even amid the stern despotism of the European states, empiricism is found flourishing, side by side, with medicine in its most scientific, most improved shape. England has been called the "*Paradise of Quacks;*" and yet medicine has been liberally patronized by the British government, while, for many centuries past, there have flourished there some of the great lights of the profession. There have lived physicians the most erudite and accomplished—of attainments so varied and profound, that the great scholar, Parr, was compelled to concede the palm of learning to the profession of which they were members. But in the midst of these medical philosophers, and in the shade of their ancient and richly endowed universities, the empiric has reared his standard and attracted his host of believers. Was it not, indeed, on that island, and among that nation of savans, that Mesmerism gained its strongest foothold and exercised the widest dominion? Men appear to have an organ of *gullibility.* Humbuggery prevails in every trade and profession. Nothing is so sacred as to be beyond its reach; nothing so humble that it will not descend to it. It has prevailed in theology and in medicine, in politics and in education. Animal magnetism has served to lead astray the gifted and the scientific, as ghosts, witchcraft, the hazel rod, and seventh son, have, time immemorial, been the ignes fatui of the vulgar.

But, I repeat, because we cannot, all at once, cut this monstrous evil up by the roots, it is bad policy to surrender the Society, and give over our efforts at improving the medical profession. This is a distemper of the public mind for which our art has no specific. In the management of it we must rely upon "general principles." It is of that class of disorders which we wish to see our patient out-grow, or which must be cautiously and gradually worked out of the system by a wise course of hygiene. I regard it as a point settled, that we cannot suppress quackery by any force likely to be granted by the legislature of the State; but it is in the power of the profession, acting in a body, and with wisdom, to elevate medicine to a point where it will not be affected by empiricism. And to this point it is the tendency of this Society to raise it in the State. It has already carried the fame of some of its members beyond the limits of the commonwealth. Many papers read at its meetings have been printed, and have found their way into the medical journals of the country. Much matter has, thus, been brought before the public which, else, had never seen the light; and, in this way, have been communicated to the world facts which will aid to swell the mass of medical learning and experience. Above all, the Society has quickened the energies and fired the ambition of many physicians who will henceforth labor with redoubled zeal for the promotion of medical science. It has now been eleven years unintermittedly in operation. Its meetings have sometimes been thinly attended, but not a year has passed without the assembling of a portion of its members, the reading of a number of papers, and the publication of transactions. The medical topography of several regions of the State has been given to the world, and there can hardly be a doubt, that the physicians who were engaged in investigating the sources and nature of the diseases of their neighborhood have thereby become better practitioners.

This appears to me the true path in which the Society ought to progress. Its literary exercises are its vital principle. They are its circulating fluid, and when they cease, the Society, for all useful purposes, may be pronounced dead. They give to it all its interest, all its influence abroad, and all its value in the estimation of men. Upon these must rest its claims to the gratitude and respect of other generations. It speaks well for the perseverance of its members, that, in a region where so many enterprises are set on foot, and so few pursued to a successful completion, it has continued to hold its meetings without interruption for so many years.

The influence of the Society upon medical education will be salutary, and this is one of the objects of which it should never lose sight. It is, perhaps, rather a fashion with gentlemen whose own literary attainments are not remarkable to declaim against the slender qualifications of their brethren, and clamor for a more thorough preparatory education. So far as my acquaintance goes, it is among those whose own preliminary training has been most deficient that this complaint is loudest. None, it is said, look with so much indulgence on the "fears of the brave and follies of the wise," as those who are themselves wise and brave. It might not be amiss for those gentlemen who are so clamorous for high literary excellence, to remember the case of the illustrious John Hunter, who was never able to write correctly his own mother tongue, and knew no other, but who, as an anatomist, physiologist and surgeon, was the glory of his own times, and through all time will be regarded as one of the great luminaries in medicine;—a man who drew his information directly from nature, and studied the laws of the animal economy and of disease in his dissecting room, in hospitals, and upon the living systems of the inferior animals. He went at once from the carpenter-shop of his brother-in-law to the anatomist's table, and without any of the aid which learning affords, not only reached the head of his profession, but took up the science where his predecessors had left it, and enriched it with more valuable discoveries than a whole generation of common minds could do. I might also cite the example of another surgeon whose fame is scarcely less than that of John Hunter. I mean Sir Astley Cooper, who likewise entered upon the study of his profession without much preparatory education, and never made pretensions to scholarship. Since I wrote this line I have read that he, too, is numbered with the great departed. Full of years, honor and riches, he has been called away from the field of his brilliant triumphs. For half a century he stood confessedly at the head of his profession in the great British metropolis, performing every great surgical operation, lecturing to large classes of eager pupils, and amid all his labors finding time to compose splendid works, the result of his own observation and experience, but, all the while, comparatively negligent of the style in which his thoughts were conveyed.

But, while the eminence to which these giants in our profession attained, in spite of the deficiences of their early education, ought to mitigate the severity of those gentlemen who bear so hard on physicians not having the good fortune to be skilled in classical learning, it is far from my intention to intimate, that their example is one to be imitated, in this neglect of early culture, or to depreciate thorough mental discipline and finished scholarship as helps to the acquisition of medical knowledge, and as accomplishments which have reflected upon our profession a glorious lustre. "*Learning*," as it is expressed in that curious book, "the Doctor," "*is a brave thing*." We cannot have too much of it. But it is the "skill of the physician," according to the son of Sirach, "which shall lift up his head, and in the sight of great men cause him to be in admiration." And the history of Hunter and Cooper, not to name others, and not to come nearer home, may teach us, that consummate skill is attainable in our profession without the aid of Greek or Roman learning, without the study of Hippocrates in the original, and even without a mastery of our own vernacular tongue! Nevertheless, let us frankly acknowledge our deficiences. I believe every physician in the country deplores the limited attainments of too many of the profession, and admits that the general standard is too low. As a profession we are in the habit of neglecting polite literature, and what is far worse, have been satisfied with very shallow draughts at the spring of medical learning. It is a great error in our system of medical education, that students are permitted to enter upon the practice of physic after too brief a term of pupilage; for although I agree that the tedious novitiate which the ancient system imposed may be abridged with advantage, since Hippocrates is not to be translated nor Cullen's Outlines committed to memory, still I must contend that "*six weeks' study, off and on,*" is too short a period to indoctrinate even the aptest pupil in all the mysteries of our comprehensive and most difficult art. Five years was the term formerly required. Dr. Rush studied five years; but he thought, and was in the habit of saying, that, so much had the introduction of principles in medicine simplified it, the students of his day could accomplish more in three years than he was able to do in five. No doubt this is true. No doubt that in three years one may acquire more valuable medical knowledge than students did in thrice three years under the tuition of Boerhaave, or even of Cullen. But if medicine has been so much simplified, it has also been greatly extended. Facts have been multiplied in every department of it, and new branches have been developed. How vastly, for example, has the range of chemistry been widened since the time of Boerhaave, and even since Rush was a teacher of it, and Pathological Anatomy, General Anatomy, Hygiene and Medical Jurisprudence may be said to have been created since the time of the great teacher of Leyden. That it is possible, in the short term devoted to study by a majority of young men before taking their degrees, to compass a knowledge of these branches, and of all the other parts of medical science, it is preposterous to suppose. The most that can be attained in that time is a general bird's eye view of the whole field, with a knowledge of the more practical departments sufficiently minute to enable the young practitioner to pursue his calling with reputable success. If he would rise to eminence in his profession—if he aspire to the rank of a learned and accomplished physician, he must continue his studies faithfully during his whole career.

The primary education of young men designed to pursue medicine as their profession, I have admitted, is not in every case as finished as could be desired. And this is one of a class of crying sins of all this region of our great country—a neglect of the early education of our sons. The South-Western and Southern States have long slumbered in regard to this great concern, and, if they would maintain their rank among civilized nations, must arouse from their lethargy. I believe they are already waking up. I believe the evil is beginning to be felt seriously, and this is a material step towards its correction. But until the whole system is improved, it is vain to hope that students of medicine will be prepared for the profession by a judicious and complete course of preliminary training.

But, supposing the early advantages of the student to have been all that is desirable, the time usually devoted to medical study is too short. Many young men obtain the degree of M. D. after less than two years' study. In such instances, no doubt the application of the pupil has been good; but however studious he may have been, and however satisfactory the examination he is able to pass, it must, nevertheless, be admitted that the knowledge he has acquired in this time is comparatively crude, and his experience in all that relates to the practice of medicine in its several departments quite too limited for the exigencies that he goes forth to encounter. The simplest operations and processes of the art must be practised by the pupil before he is prepared for the duties of his profession, and for this he must have time as well as opportunity. There must be patients to be examined and administered to, either with his private preceptor or in the wards of a hospital. Time is necessary for this; he cannot be engaged with his books while he is studying disease at the bed side, and yet much reading is indispensable as a preparation for this exercise, for he ought to be well grounded in the principles of medical science before he engages in the investigation of the actual phenomena of disease.

But I have yet to mention the defect, in the system of medical education too generally pursued in the Western country, which is doing most to retard the march of medicine to the high place in the public confidence which it is destined to attain. The elementary education of students of medicine is too limited. We grant this, and hope, as the country advances in wealth and intelligence, that this complaint will cease. The period of professional study, we have also admitted, is too brief, and the medical schools of the country, aided by the private practitioners, it is to be expected will ere long correct this evil. But that which is the sorest evil is not so much these, as the engaging of so many young men in the practice of physic after a short course of reading, without visiting any medical school, and, consequently, without any practical knowledge of anatomy, healthy or morbid, and with but little of operative surgery. The influence of this upon the profession can easily be imagined. Such practices inevitably sink it in the public estimation, and tend to bring every class of practitioners to the same level. The population of Tennessee is greater, by one third, than that of Vermont, its climate less salubrious and the number of its physicians probably twice as great. Yet, during the last winter, according to the printed catalogues, Vermont had as many of her sons in attendance on medical lectures as Tennessee. The inference is, that, in the latter state, very many assume the responsibilities of an art involving human life without those advantages which are now admitted, on all hands, to be necessary to the acquisition of a competent knowledge of medicine. If I am mistaken as to the extent and magnitude of this evil, no one will deny that it has an existence in the state, any more than that the correction of it is demanded alike by the dignity of the medical profession and the best interests of society.

Since I have engaged in the task of enumerating the defects which attach to our profession, and which it is one of the purposes of this Society to remedy, I must not pass in silence over one which attaches to the Medical Schools in the United States. I allude to the shortness of the lecture term. In every school of distinction the number of chairs is from six to eight. Anatomy, Surgery, Chemistry, Materia Medica, Theory and Practice, and Obstetrics are taught in all; and in many there are chairs, also, of the Institutes of Medicine, Pathological Anatomy, and Medical Jurisprudence. And yet the period devoted to delivering a full course of instruction on all these subjects is but four months. The consequence is that the lectures are too much crowded, six a day being common, and, in some schools, seven or eight each day in the week, Sabbaths excepted, while, it is plain, the lecturer must be straightened for time and hurry over many important subjects. If this were the fault of one school, or of a few, it might the sooner be remedied; for students could then resort to those in which more time was given for arranging and assimilating the matter thrown out by teachers, and more leisure allowed for attendance upon hospitals and the anatomical rooms, now universally regarded as the most profitable sources of medical instruction. But it is one which attaches to all alike. In none is the regular term extended beyond four months, while in some it is not even so long. This being the fact, it becomes a difficult matter to apply the corrective, no school venturing to take the lead lest the additional expense imposed upon pupils by their longer absence abroad should deter some from attending it. The proverbial impatience, too, of American young men—their haste to rush "*in medias res*," and complete the irksome period of pupilage militates against such a change. It is with difficulty that a respectable portion of them can be prevailed upon, during the first winter of their attendance, by the entreaty of professors, and by all the terrors of the "*green box*," to remain to the end of even our short sessions; and not a few hurry off, from all the schools with which I have had any acquaintance, by the first of February, thus throwing away the benefit of one fourth of the whole course; as if their sole object in leaving home were to get the name of attending lectures. In this way they are doing much to detract from the reputation of medical schools, and to bring down the character of the profession. The schools, most unwisely as I conceive, adhere to their short sessions, and students, impatient of labor, curtail them still further; so that, practically, for very many of those who attend but one course, the term is reduced to one quarter of a year. And with the knowledge of man's complicated system and of the diseases which assail it, with the slender outfit derived from this three months' study at a medical school, and a few months' previous reading, do many young men launch out upon the perilous voyage of life—perilous alike to their own reputation, and the health of those who confide in their skill. There is a solemn duty which lies before us here, and equally before the private preceptor and the public teacher. The practitioner, when he sends his pupils to a medical school, ought to charge them to remain until they should hear the last word of the valedictory. Every father should exhort his son to hold out to the end, and with this aid from a quarter so influential, professors would be able to maintain an undiminished class to the end of the term.

Having now stated with candor what I deem defects in our present system and the remedies to be applied, I will declare my belief, that if students avail themselves, in good faith, of all the facilities for the acquisition of knowledge which the American schools of medicine afford, notwithstanding the courses be too short and the lectures too crowded, they prove when they enter upon the active duties of their profession as energetic and successful practitioners as are turned out by the schools of Europe. In the hospitals, which are now found in connexion with nearly every medical institution, they have opportunities of witnessing many of the surgical operations that are likely to fall in their way, and of observing the symptoms and treatment of disease under teachers of learning and experience; while, in the dissecting room, if they be diligent, they may make themselves practical anatomists, and acquire the mechanical tact that will fit them for the duties of the surgeon. Under this system, there are young physicians every year coming forth, from all our schools, who, for judgment in diagnosis, and skill in the management of disease, would compare with the élèves of Paris or London. I speak thus confidently because I have had the most repeated opportunities of comparing the success of

American graduates with that of young practitioners, who have visited the medical institutions of Europe, and because medical statistics bear out the assertion. It is in no spirit of overweaning national vanity that I affirm that I have not found the graduates of London, Paris, or Edinburgh superior, at the bed side, to graduates of the American schools of equal capacity, who have devoted themselves with equal assiduity to their studies. That in general information and literature, and also in a knowledge of pathological anatomy, those of foreign education may have the advantage, I will not deny; but in their ability to treat disease they have in no wise manifested a pre-eminence over those who have studied medicine at home. The medical statistics of France, where the science may be considered as at its highest point, afford no ground of boasting to our French brethren over the physicians on this side of the Atlantic. Better anatomists we concede them to be; in pathological anatomy, particularly, we yield the palm to them, as must the profession of every other nation—that they are cultivating all the branches of medical science with greater industry and with higher success, and are taking the lead of all the world in toxicology, physiology, and pharmacy, we readily admit; but, after all, when it comes to the application of the principles—"to war with death and stop his flying dart," the statistics of the two countries show, that the practitioners of the Mississippi Valley cure as large a proportion of their patients, as the pupils of Louis or Broussais. I will go further and say, that the surgeons of this region have enjoyed a degree of success in their operations, especially in that for stone in the bladder, quite unrivalled by anything that the history of the hospitals of London or Paris can adduce. I do not pretend to affirm that the fact is proof of superiority of skill in Western surgeons, but it is one which justice requires to be stated, and I think it must be admitted, that students are not badly taught when the practitioners are proving themselves equal to the highest functions of their profession.

The practitioners of the South-west are wedded to no theory in medicine; for if they have, at different times, been carried away by the elegant speculations of Rush, or the bold dogmatism of Broussais, or the simple theory of the Congestive school, their sound, good sense and innate independence of spirit have brought them back, after a temporary bondage, to a wise system of eclecticism. If they make few additions to the stock of medical knowledge, they are on the alert to catch the first dawnings of any discovery that may arise in another hemisphere. The new remedy introduced into the hospital at Dublin or Paris, in the spring, will be in the hands of the practitioners of Tennessee and Missouri before midwinter, and the new operation performed by Dieffenbach, in Germany, at the beginning of one year, will be repeated by the surgeons on the Ohio and Mississippi before the beginning of the next. So facile and rapid, in our day, is the interchange of knowledge, and so prompt the physicians of the United States to seize and apply whatever may be devised, by human ingenuity, for the relief of human suffering in any quarter of the globe.

This devotion to system to which I have referred, and from which we claim exemption—this proneness in the minds of men "*jurare in verba magistri*"—this blind belief in theory, and the perfectibility of medicine, has made sad work in the profession. For how many centuries after Hippocrates flourished were men content servilely to tread in his footsteps, assured that the science and the art of healing could be carried no farther. Then came Galen, who was but little more than a commentator on the Father of medicine—"*fimbria Hippocratis*"—but with disciples so ardent and confiding as to be ready to aver, that nature herself had changed, rather than admit the probability of their masters having gone astray in their description of the human body. And at a later day, and after some centuries of darkness, Bombastus Paracelsus arose to decry and set at naught all that Hippocrates and Galen had written concerning medicine. "The down on my bald pate," he declared—"*stultissimus pilus*"—knows more than all your doctors; the very buckles on my shoes are more learned than your Galen and Avicenna; and my beard has more experience than all your universities." Claiming to be himself "*primus medicorum*," he boasted that he made more famous cures than all the physicians in Europe besides, denounced Hippocrates and his followers as infants, idiots, and sophisters, and, seated in his professional chair, at Basil, with great solemnity, burnt the writings of Galen and Avicenna. So Thessalus, before, had written to the Emperor Nero, that he had founded a new sect, *which is the only true one.* "I have been forced to this," he says, "because none of the physicians who have preceded me have discovered any thing useful either for the preservation of health or for the cure of diseases, and because Hippocrates himself has put forward many dangerous maxims. His system was a very simple one, leaving but little to the discrimination of the physician; namely:—that nature, in each case, points out to the patient what is most fit for him, and that, hence, he should be diligently supplied with every thing that he may fancy.

But, anon, Hahnemann appears, to found still a newer school, and put forth a better system of cure, and like his predecessors, in the same line, he must needs begin by denouncing the labors of others as vain and unprofitable. In allusion to the Hippocratic Medicine, he says, with an arrogance which the prince of quacks himself could scarcely exceed, "since this art only consists in a gross imitation of a dangerous and insufficient process, it must be admitted that the *true* medicine was not discovered *till by me.* It is the infallible oracle of the art of curing; it is the sole mode of really curing disease, because it reposes on an eternal and infallible law of nature." And what are these vaunted principles? Truly, these: that it is absurd to seek for the cause of symptoms in order to remove them! that we must cure diseases by the exhibition of medicines which would produce them in a healthy person; that the dose should be infinitely small; and that there are but three original diseases from which spring all the maladies that afflict mankind, namely: "syphilis, sycosis and the itch," corresponding to "the oil, the spirit and the salt," of Paracelsus, "which burn the patient as hell does."

Nor was Broussais, with all his genius, learning and real merit, much more modest in the annunciation of his doctrines —the physiological medicine. "After so many vacillations in its march," says he, "medicine at length follows the *only path which can conduct it to truth*—the observation of the relations of man with external modifications, and the relations of the organs of man one to the other." "What," he exclaimed, in the words of the great Bichat, "is observation, if we are ignorant of the seat of disease?"

This is the language of the *Anatomical School* of physicians, who, with the Hippocratists, divide the Faculty of France. With them, all that had been observed and experienced from the earliest times of medicine down, passes for nothing. Nothing has value with them which the dissecting knife does not reveal or sanction. This is their *experimentum crucis*, the universal test to which every phenomenon in disease must be submitted; and with a fate harder than that of the blind poet, "with knowledge from one entrance quite shut out," they obstinately refuse to receive intelligence through every avenue but one. Symptoms, according to this school, are hieroglyphical until numerous autopsies have deciphered them, and all cures are empirical, and at best but fortuitous, unless made in accordance with the principles of pathological anatomy.

Happily, we are committed to none of these systems. In the spirit of that freedom which our institutions breathe, we welcome truth from whatever clime she may come, doing justice to all schools, forming entangling alliances with none. We are all Hippocratists; we are all Physiologists. We are even willing to be so far Homœopathists, as to give strychnia in paralysis, and bark in intermittent fever. We hail pathological anatomy as a noble branch of Medical science, and honor our French brethren for the zeal with which they have cultivated and enriched it; but in doing so, we have not learnt to despise all that our fathers treasured up in Medicine of sage experience and enlightened observation. While we glory in the advances which the healing art has made, and is destined to make, in the path that Bichat pointed out, and Broussias pursued with so much success, we cannot forget, that without any knowledge of the pathology of Small Pox, Sydenham declared the true principles of its treatment; we cannot forget that it was observation, not to call it accident, which placed in our hands the prophylactic of this loathsome disease, and we are obliged to own, that by the same process we came in possession of Peruvian bark, opium, antimony, and mercury. We cannot, therefore, agree to discard observation and experience as among the instruments by which the noble fabric of our art is to be reared and carried towards perfection. We do not profess to be giants in Medicine, but standing as we do upon the shoulders of these giants of a former time, we suspect that we may be able to see farther and more clearly than they.

*May* 5, 1841.

